# Supplementary material for: Identifying environmental factors associated with tetrodotoxin contamination in bivalve mollusks using eXplainable AI
Source: NPJ Sci Food. 2026 Apr 17;10:192. doi: 10.1038/s41538-026-00848-x (PMC13273195; doi:10.1038/s41538-026-00848-x)
Supplement: Supplementary file 1 — 41538_2026_848_MOESM1_ESM [file 41538_2026_848_MOESM1_ESM.pdf]

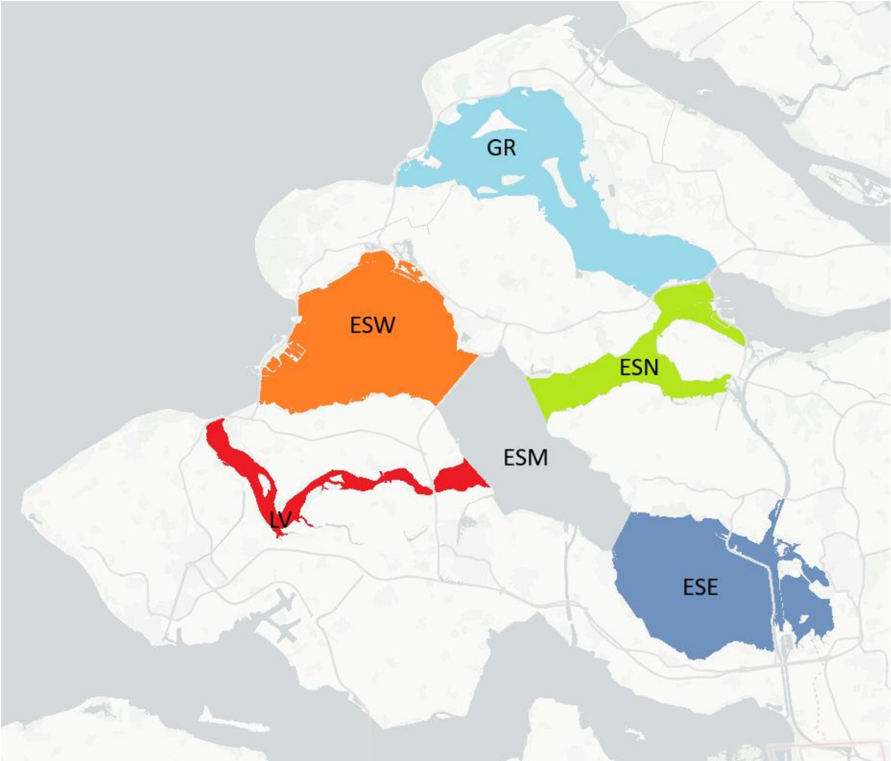

Supplementary Figure 1: Measurement regions in Zeeland. The regions are the Eastern Scheldt east (ESE), north (ESN), west (ESW), middle (ESM), Lake Grevelingen (GR), and Lake Veere (LV). Tetrodotoxin (TTX) was detected in a total of 222 samples and no TTX in 934; the number of samples for each region are ESE (N=222), ESN (N=204), ESW (N=207), ESM (N=208), GR (N=167), VM (N=148).

Supplementary Table 1: Used hyperparameters for model development. The optimal and final parameters are selected based on validation Area Under the Curve (AUC) score.

**Hyperparameter**	**Search Space**	**Final model**
LSTM Layers	1-5	3
Batch size	16/32/64	32
Learning rate	0.001-0.00001	0.0005
Dropout	0.2-0.5	0.3
Hidden dimension	64-128-256-512	256
Learning rate decay	NA	0.1
Step size	NA	30

Supplementary Table 2: Mean (standard deviation) of the 35-day average of the meteorological and hydrological features. The significance of each univariate feature between "No TTX" and "Above AL", each significant feature has been bolded. To consider significance a Bonferroni corrected p-value of 0.002174 was used.

**Feature**	**ALL (N = 1,156)**	**Above AL (N = 75)**	**Above LL (N = 44)**	**No TTX (N = 934)**	**P-value**
Week number – (1-52)	27.6 (± 10.4)	24 (± 2.08)	23.4 (± 1.56)	28 (± 10.4)	0.060057
Sunrise – hour	5.21 (± 1.33)	3.86 (± 0.26)	3.79 (± 0.13)	5.30 (± 1.33)	**0.000001**
Sunset – hour	18.78 (± 1.41)	20.24 (± 0.25)	20.29 (± 0.10)	18.68 (± 1.41)	**0.000000**
Sun hours – number of hours	13.57 (± 2.74)	16.38 (± 0.50)	16.50 (± 0.23)	13.37 (± 2.72)	**0.000000**
Global radiation – J/cm^2	69.89 (± 24.11)	88.15 (± 19.17)	90.20 (± 19.06)	68.62 (± 23.91)	**0.000000**
Temperature – °C	15.06 (± 4.8)	17.09 (± 1.26)	16.93 (± 1.14)	14.92 (± 4.93)	0.108175
Max temperature – °C	18.32 (± 5.39)	20.88 (± 1.54)	20.79 (± 1.46)	18.14 (± 5.52)	**0.001176**
Min temperature – °C	12.18 (± 4.41)	13.83 (± 1.15)	13.62 (± 0.95)	12.06 (± 4.52)	0.866331
Water temperature - °C	16.18 (± 5.19)	18.24 (± 1.49)	18.00 (± 1.25)	16.04 (± 5.32)	0.099688
Water height - cm	2.04 (± 6.93)	0.23 (± 5.21)	-1.18 (± 4.78)	2.16 (± 7.02)	**0.000187**
Water height calculated - cm	1.82 (± 5.13)	-2.38 (± 2.53)	-3.38 (± 2.16)	2.11 (± 5.14)	**0.000884**
Mean wind speed – m/s	3.51 (± 0.48)	3.21 (± 0.18)	3.18 (± 0.18)	3.53 (± 0.49)	0.861240
Average wind direction - °	174.9 (± 63.7)	194.7 (± 63.9)	194.4 (± 66.4)	173.6 (± 63.5)	**0.000002**
Ph	8.20 (± 0.15)	8.24 (± 0.09)	8.24 (± 0.08)	8.19 (± 0.15)	**0.000044**
Oxygen concentration – mg/L	8.77 (± 1.33)	8.41 (± 0.69)	8.48 (± 0.57)	8.80 (± 1.36)	0.820732
Oxygen Saturation %	101.2 (± 7.16)	101.9 (± 5.83)	102.2 (± 5.25)	101.1 (± 7.24)	0.052270
Chlorophyll concentration - µg/L	4.98 (± 3.27)	3.93 (± 2.11)	3.20 (± 1.21)	5.05 (± 3.32)	0.825772
Pheophytin concentration - µg/L	0.12 (± 0.06)	0.12 (± 0.06)	0.10 (± 0.05)	0.12 (± 0.06)	0.483759
Air pressure - hPa	1016 (± 5)	1014 (± 5)	1014 (± 5)	1016 (± 6)	0.447946
Chlorosity – g/L	13.05 (± 4.80)	15.35 (± 2.35)	15.69 (± 1.74)	12.89 (± 4.89)	0.026509
Chloride concentration – mg/L	9.95 (± 7.52)	7.49 (± 7.82)	8.66 (± 7.91)	10.12 (± 7.47)	0.002238
Conductivity – mS/m	2971 (± 1141)	3675 (± 597)	3741 (± 450)	2922 (± 1153)	0.320361
Salinity - g/l	29.74 (± 1.82)	29.31 (± 1.37)	29.54 (± 0.97)	29.77 (± 1.85)	0.039168

Supplementary Table 3: Number of samples in the train, validation, and test sets. The total number of samples is the sum of the samples with 'no TTX' and 'above LOD'. With an additional distinction for samples with tetrodotoxin (TTX) above Action Limit (AL), Limit of Detection (LOD), and Legal Limit (LL). For the LOD and LL, no training was carried out.

	**Years**	**Total**	**No TTX**	**Above LOD**	**Above AL**	**Above LL**
Train	2016-2021	857	726 (85%)	131 (15%)	47 (5%)	25 (3%)
Validation	2022	155	118 (76%)	37 (24%)	13 (8%)	9 (6%)
Test	2023	144	90 (63%)	54 (38%)	15 (10%)	10 (7%)
Total	2016-2023	1156	934 (81%)	222 (19%)	75 (6%)	44 (4%)

Supplementary Table 4: Comparative trials of the LSTM model with classical machine learning approaches. The LSTM method is compared by the following algorithms: support vector machine, random forest, and XGBoost. Our LSTM method outperforms each approach both in AUC (minimum of 0.04 difference) and specificity (minimum of 0.13 difference). The specificity were calculated at a threshold of 90% validation sensitivity.

	**AUC**	**Specificity @ 90% sensitivity**
SVM	0.79	0.18
RF	0.89	0.52
XGBoost	0.87	0.68
LSTM	0.93	0.81
